# High Frequency of Either Altered Pre-Core Start Codon or Weakened Kozak Sequence in the Core Promoter Region in Hepatitis B Virus A1 Strains from Rwanda

**DOI:** 10.3390/genes10030182

**Published:** 2019-02-26

**Authors:** Heléne Norder, Theogene Twagirumugabe, Joanna Said, Yarong Tian, Ka-Wei Tang, Lars Magnius, Magnus Lindh

**Affiliations:** 1 Department of Infectious Diseases, Institute of Biomedicine, Sahlgrenska Academy, Gothenburg University, 405 30 Gothenburg, Sweden; joanna.said@microbio.gu.se (J.S.); yarong.tian@gu.se (Y.T.); kawei.tang@gu.se (K.-W.T.); magnus.lindh@microbio.gu.se (M.L.); 2 School of Medicine and Pharmacy, College of Medicine and Health Sciences, University of Rwanda, Kigali, Rwanda; twagirumugabe@gmail.com; 3 Ulf Lundahl Foundation, 100 61 Stockholm, Sweden; lars.magnius@gmail.com

**Keywords:** HBV, basal core promoter, subgenotype A1, Sub-Saharan Africa

## Abstract

Hepatitis B virus (HBV) is endemic in Rwanda and is a major etiologic agent for chronic liver disease in the country. In a previous analysis of HBV strains from Rwanda, the S genes of most strains segregated into one single clade of subgenotype, A1. More than half (55%) of the anti-HBe positive individuals were viremic. In this study, 23 complete HBV genomes and the core promoter region (CP) from 18 additional strains were sequenced. Phylogenetic analysis of complete genomes confirmed that most Rwandan strain formed a single unique clade, within subgenotype A1. Strains from 17 of 22 (77%) anti-HBe positive HBV carriers had either mutated the precore start codon (9 strains with either CUG, ACG, UUG, or AAG) or mutations in the Kozak sequence preceding the pre-core start codon (8 strains). These mutually exclusive mutations were also identified in subgenotypes A1 (70/266; 26%), A2 (12/255; 5%), and A3 (26/49; 53%) sequences from the GenBank. The results showed that previous, rarely described HBV variants, expressing little or no HBeAg, are selected in anti-HBe positive subgenotype Al carriers from Rwanda and that mutations reducing HBeAg synthesis might be unique for a particular HBV clade, not just for a specific genotype or subgenotype.

## 1. Introduction

Hepatitis B virus (HBV) is a DNA virus of the family of Hepadnaviridae. It has a compact genome of about 3.2 kb with a genetic variability classifying the strains into nine genotypes (A-I) [1,2,3,4], each further subdivided into subgenotypes. Subgenotypes A1, A3–A7, D1, D2, D4, and D6–D8, as well as genotype E are prevalent in African countries [5,6], with subgenotype A1 being the prevalent HBV type in Rwanda [7].

The HBV genome has four overlapping open reading frames (ORF), the preS1/S2/S coding for surface antigens, the P encoding the polymerase, the precore/C encoding for both the circulating e-antigen (HBeAg) and the core antigen, and the X ORF encoding the HBx protein. The genome also has four promoters (preS1, preS2, core, and X) and two enhancer elements (ENI and ENII) located upstream of the core promoter [8]. There are seven polyadenylated and capped viral RNA transcripts which encode the viral proteins [9,10]. The pregenomic RNA (pgRNA), which serves as a template for reverse transcription into HBV DNA also encodes for both the core and the polymerase proteins. HBeAg, discovered in the early 1970s [11], is a suggested T-cell tolerogen [12,13], and is produced from the translation product of the longest HBV mRNA with transcripts initiating 29 codons, upstream of and in frame with the C start codon [8]. HBeAg is secreted from the hepatocyte into the blood and is a marker of active ongoing replication. Seroconversion to the corresponding antibody, anti-HBe, is often a sign of remission. Some patients, however, show persistence of serum HBV DNA, despite seroconversion to anti-HBe, due to the emergence of mutations in the core promoter or precore region. 

The core promoter ((CP); nt 1575–1849) has an important role in the replication of HBV. It consists of the basal core promoter ((BCP); nt 1743–1849), partly overlapping with the precore region, and the upper regulatory region ((URR); nt 1613–1742). The BCP is sufficient for accurate initiation of both the precore-mRNA and pgRNA transcription, in vivo, and it contains the direct repeat 1 region, which is required for reverse transcription. The CP, thus, regulates transcription of the precore-mRNA and hence mutations in this region might cause reduced levels of HBeAg expression [14,15,16]. Therefore, certain mutations in this region can affect HBeAg synthesis without adversely affecting the ability of HBV to replicate [15,17,18]. The most common mutations in BCP are double mutations, at nucleotide positions 1762 and 1764, A1762T and G1764A, which are associated with downregulation of the production of HBeAg [15,18]. These double mutations have been detected more frequently in patients with fulminant hepatitis, than in asymptomatic carriers [19]. Other CP mutations have been shown to affect viral DNA replication and HBeAg expression [20]. Mutations C1766T and/or T1768A have been shown to enhance pgRNA synthesis 2.5–5-fold and reduce the HBeAg synthesis, at the same magnitude, by downregulating the precore mRNA [21]. Insertions within BCP causing increased pgRNA synthesis have been described in patients with chronic and fulminant hepatitis [22,23].

Mutations in the precore region are often nonsense or cause a frameshift which might terminate the HBeAg expression [24,25]. The most common is a G1896A mutation that forms a stop codon (TAG), within the precore region, thus, abolishes the translation of HBeAg [25,26]. This precore stop codon appears exclusively in strains with thymidine at nucleotide position 1858, thereby, not in genotype A, F (except subgenotype F1), and subgenotype C2, which express a cytosine at this position. All genotype G strains have two stop codons in this region, at positions 2 and 28. The double BCP mutations and precore stop codons are not mutually exclusive [16]. Lack of HBeAg expression can also be due to different mutations in the translation initiation codon of the precore protein. However, these variants seem to be infrequent and have previously only been described in isolated strains [19,26,27,28].

This study investigated mutations in the HBV genome that might explain the high frequency of viremic infections in anti-HBe positive carriers in Rwanda.

## 2. Materials and Methods

### 2.1. Serum Samples

Serum samples from 25 Rwandan individuals with no known liver disease (20 blood donors and 5 healthy individuals) and 16 Rwandan patients with liver disease were used in the study. The patients were attending liver disease clinics at six different hospitals, from all regions of Rwanda. The patients were diagnosed with either chronic hepatitis or cirrhosis. The persons designated as healthy did not know that they had any liver disease. Determinations of HBeAg and anti-HBe, and sequencing of the S-genes, in all samples, have been described previously and are shown in Table 1 [7,29]. The viral load in the sera has also been described previously [7,29].

The study was approved by The Rwanda National Ethics Committee (RNEC 024/2014).

### 2.2. PCR Amplification

Nucleic acid extraction and virus DNA amplification were performed, as described previously [7]. Complete HBV genome could be amplified for 23 strains (Table 1). The primers used are given in the Supplementary Table S1.

Partial CP region could be amplified with the same primers as those used for complete genome sequencing in additional 18 strains (14 from carriers with anti-HBe, 3 from persons with HBeAg, and one from a patients with liver disease, lacking markers for HBeAg and anti-HBe).

### 2.3. Sequencing

All amplified PCR products were purified and extracted with QIAquick PCR Purification Kit (Qiagen, Hilden, Germany), according to the manufacturer’s description. The purified products were cycle sequenced in both directions, using 1.6 μM of the same primers as in the PCR, in the BigDye Terminator Cycle Sequencing Ready Reaction kit (Applied Biosystems, Carlsbad, CA, USA), according to the manufacturer’s instructions. The sequences were obtained by the 3130 × l Genetic Analyzer (Applied Biosystems).

### 2.4. Phylogenetic Analysis

The sequences obtained were analyzed in the SeqMan program in the DNAStar programme package version 10.1.2 (DNA Star Inc, Madison, WI, USA). The sequences were aligned with 526 complete genomes, representing all HBV genotypes, obtained from the GenBank, including 75 genomes from subgenotype A1 strains from Africa. Phylogenetic analysis was carried out with the PHYLIP package version 3.65. Evolutionary distances were calculated using the F84 algorithm in the DNADIST program, with a transition/transversion ratio of 1.34, with gamma correction and alpha 0.23. Phylogenetic trees were constructed using the unweight pair-group method, using arithmetic averages (UPGMA) and the neighbor-joining method in the NEIGHBOR program in the PHYLIP package. Bootstrap analysis for 1000 replicas was performed with the SEQBOOT and CONSENSE programs in the PHYLIP package. The sequences obtained in this study are deposited in GenBank with accession numbers MK512455-MK512477.

## 3. Results

### 3.1. Complete HBV Genomes

**Figure 1:**
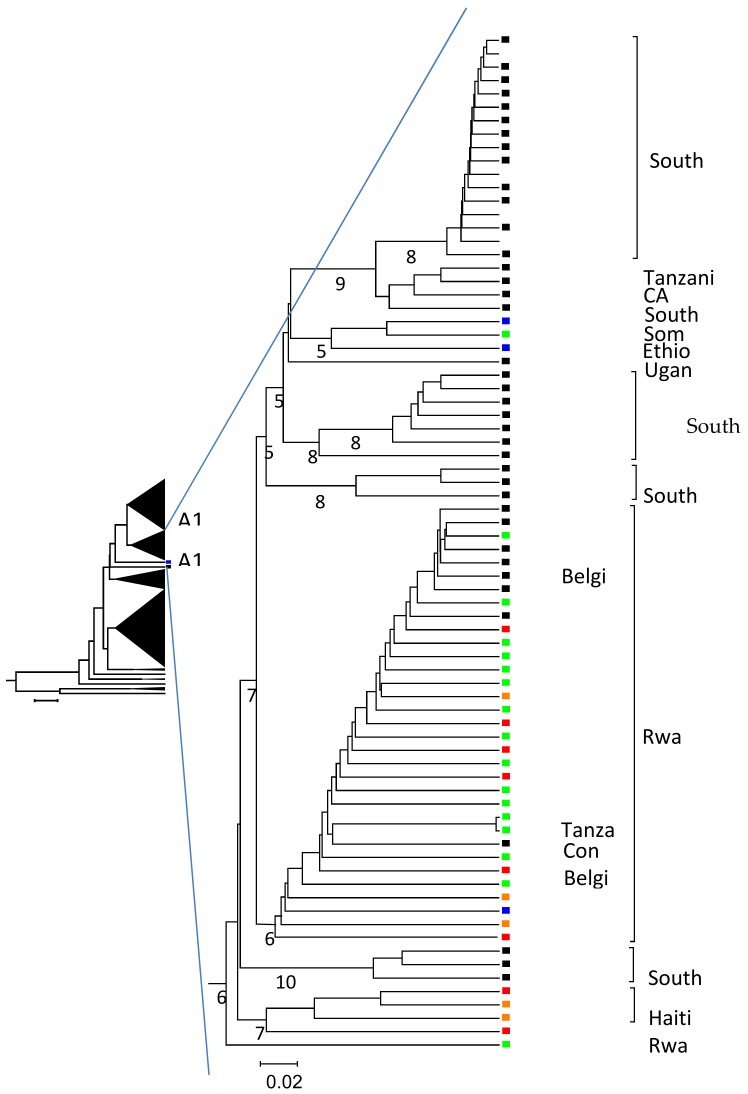
Branch from an unweight pair-group method, using arithmetic averages (UPGMA) tree based on 526 complete Hepatitis B virus (HBV) genomes. The clades formed by each subgenotype of A are shown in the small tree to the left. One of two branches formed by subgenotype A1 complete HBV genomes, is enlarged. The origin of strains from the same country is marked with brackets at the nodes, the origin of the other stains are given at the nodes. Strains with wild type precore start codon and Kozak sequence preceding the precore start from patients with unknown HBeAg/anti-HBe status are marked by black squares at the nodes. Strains from HBeAg positive patients are marked by green squares. Strains from patients with anti-HBe and wild-type precore start codon and Kozak sequence are marked by blue squares. The strains marked with red or orange squares have either a changed precore start codon (red) or changed Kozak sequence (orange). The figures below the branches refer to boot strap values of 1000 replicas.

Complete genomes were obtained for 23 HBV A1 strains. Twelve were from HBeAg positive, 8 from anti-HBe positive, and three from individuals with both HBe markers. Phylogenetic analysis of the complete genomes confirmed that most HBV strains from Rwanda, together with two strains from the neighboring Tanzania and one from Democratic Republic of Congo formed a single clade, within subgenotype A1 (Figure 1).

The P gene was 2484 nucleotides long, for all strains, apart from nine, which had deletions in the spacer region (Supplementary Table S2) [30], and one strain, RW2079, with an additional 18 nucleotide deletion between the nucleotide residues 1573–1590, in the RNaseH region of the polymerase [30]. The amino acid Gln334 in the polymerase, which is unique for subgenotype A1 [31], was present in all strains, however, the other unique A1 amino acid Lys338 was lacking in two strains, Rw14-25 from a blood donor and Rw2086 from a patient with liver disease. Both these strains expressed Gln338.

The S genes of the sequenced strains have been described previously [7,29]. The core region was 558 nucleotides long for all strains, except strain rw14–03, which had a C2396T mutation forming a stop codon, which made the core protein for this strain shorter by two amino acids, compared to the other sequenced strains (Supplementary Table S2).

The X gene was 465 nucleotides long for all strains, except three. Two had mutations affecting the basal core promoter. Strain Rw14-220 had a 12 nucleotides insertion with a stop codon between nucleotide positions 1766 and 1777, truncating the X gene to 408 nucleotides. The other strain, rw2199, had a 426 nucleotide long X gene, due to 39 nucleotide deletion between positions 1736 and 1775. The third strain with a shorter X gene, Rw2079, had the above mentioned 18 nucleotide deletions between nucleotides 1573 and 1590 also affecting the P gene (Table S2).

### 3.2. Core Promoter and Precore Regions of Complete and Partial Genomes

Due to mutations observed in the CP region in the 23 complete genomes, the region between nucleotides 1747 and 1927 was sequenced in additional 18 strains (Table 1).

The most common mutations found in 22 strains from anti-HBe positive individuals in this study, were mutations altering precore start codon, in nine strains (41%; Table 2 and Table 3) or weakening the Kozak sequence preceding the precore start codon [32], in eight strains (36%; Table 2 and Table 4). The mutations altering the precore start codon were CUG, UUG, AAG, and ACG, with CUG and UUG being the most common.

The Kozak sequence between residues 1809 and 1813 was not the typical wild-type GCACC sequence found in most genotypes, but TCATC in all strains from the HBeAg positive carriers. This Kozak sequence has also been described in strains from HBeAg and anti-HBe positive individuals from South Africa [33], and is probably the wild-type sequence for subgenotype A1 [21]. There were six different patterns of altered Kozak sequences between residues 1809 and 1813 in 8/42 (19%) sequenced strains with two Kozak sequences, TTCTC and TCCTC, shared by two strains each (Table 2 and Table 4).

None of these mutations in the sequenced strains were associated with liver disease. Among the anti-HBe positive carriers, 11 of 13 healthy persons compared to six out of eight patients with liver disease, had either an altered precore start codon or a weakened Kozak sequence. 

In the GenBank genotype A sequences, changes in the precore start codon or altered Kozak sequence preceding the precore start codon were found in strains of different origin, however, most strains with these changes were found in clades formed by strains from Rwanda, Cameroon, Haiti, and India (Figure 1 and Supplementary Figure S1). There were 29/266 (11%) subgenotype A1,7/225 (3%) subgenotype A2, and 12/49 (24%) subgenotype A3 sequences from the GenBank, with a changed precore start codon (Table 3). Changed Kozak sequences in this region were found in 41/266 (15%) subgenotype A1 in 5/225 (2%) subgenotype A2, and in 14/49 (28%) subgenotype A3 genomes in the GenBank (Table 4). The A2 genomes had other substitutions of the Kozak sequence that were not found in A1 or A3 sequences, such as ACACC, GTACC, and GTTCC.

In the BCP region, the double mutation A1762T/G1764A was observed in six of the 41 sequenced strains, three were from healthy blood donors and three from patients with liver disease. One of the strains, rw2113 from a patient, also had an altered precore start codon, and one from another patient, rw2216, had a changed Kozak sequence preceding the precore start codon.

The four regulatory regions for the TATA binding proteins, within the BCP, between nucleotides 1750–1755, 1758–1762, 1771–1775, and 1788–1795, were conserved in all sequenced strains except rw14-120, which had a T1758C mutation in the second region and strain rw2199, which had a deletion covering the first three TATA regions. These three regions are important for initiation of the precore mRNA [34]. The fourth region, important for pgRNA synthesis and the pgRNA initiation at nucleotide positions 1821–1828 [34], was conserved in all sequenced strains.

The G1888A/C mutation stabilizing the eta signal [35] was found in 27 (66%) of the 41 strains sequenced in this study. These mutations were found in strains with or without the above-mentioned mutations and from both HBeAg and anti-HBe positive individuals. None of the strains had the G1896A mutation that forms a stop codon within the precore region, thereby, terminating the HBeAg expression, or any amino acid substitution in the precore region sequenced. 

There was no correlation between viral load and the different mutations in the BCP, although the viral load in strains with altered precore start codon tended to be higher in patients with liver disease than in those without known liver disease (Table 2).

## 4. Discussion

In this study the high prevalence of viremic patients with anti-HBe without HBeAg reactivity, in Rwanda, for the majority of cases, can be explained by the identification of otherwise rarely described mutations in the CP region of the infecting HBV genomes. The most common mutations were either mutations altering the precore start codon or weakening the Kozak sequence, before the start codon of the precore gene. Both mutations, which were mutually exclusive, were likely to reduce the synthesis of HBeAg and, thereby, induce a virus escape from the host’s immune response. Mutations in the Kozak sequence have also been identified in some South African, Kenyan, and Asian subgenotype A1 strains [21,33,36]. The change of the precore start codon has rarely been described and only from a few A1 strains from South Africa and untyped strains from Japan and Europe [19,26,27,28,36]. In this study strains with these mutations were found in clades formed by A1 strains from Rwanda and Haiti, and A3 strains from Haiti and Cameroon. The clades formed by the strains with these mutations from Haiti and Cameroon were observed in the phylogenetic tree, but the mutations were not discussed in the publication describing these strains [37,38]. These results indicate that the functionally advantaged mutations reducing the HBeAg synthesis, might be restricted to a particular HBV clade and not just for a specific genotype or subgenotype.

The mutations observed in the CP region for the strains in this study, probably reduced the synthesis of the precore mRNAs but left the pgRNA synthesis essentially unaffected, since both the TATA rich region important for initiation of transcription and the initiation site for pgRNA [34] were conserved in all sequenced strains. The change of the precore start codon might abolish or just decrease the expression of HBeAg. Several of the identified mutations have been described as non-canonical start codons used by mammals, with the most efficient being the CUG [39,40], which was found in three of the strains, in this study, followed by UUG found in another set of three strains. This change of start codon for an ORF has also been found in other viruses, such as hepatitis E virus and plant RNA viruses [41,42], and has been shown to be 2–30% as efficient as AUG, as a start codon in mammalian cells [39]. CUG was the most commonly changed start codon found in subgenotypes A1, A2, and A3 sequences in the GenBank. Although the AAG has not been shown to be used as a non-canonical start codon, it was found in two strains, in this study.

The efficiency of transcription of an open reading frame is dependent on the preceding Kozak sequence with a weak Kozak sequence lowering the initiation of translation of the mRNA [42]. The strains from the anti-HBe positive persons, in this study, had either an altered precore start codon or weakened Kozak sequence changes. Possibly, the reason why the strains in this study and the sequences in the GenBank did not have both these changes, might be because both mutations might change the eta signal or that, for most strains, a low production of HBeAg might be preferential for the virus replication, which would be completely abolished if both mutations had occurred simultaneously.

One of the two most commonly described BCP mutations, the precore G1896A mutation leading to premature termination of the precore protein and, thereby, preventing the HBeAg production [25] was not observed in any strain sequenced, since they expressed C1858 [43], which stabilized the eta signal by base pairing with G1896. The G1896A mutation was, therefore, not observed in strains with C1858. The other commonly described BCP mutation was the double A1762T/G1764A mutation, in strains from viremic patients with anti-HBe [15], which occurred frequently in patients with hepatocellular carcinoma [36,44]. This double mutation was found in six strains in this study, three from healthy blood donors, and two with other BCP mutations. 

Variants with deletions of partial BCP regions need coinfection with wild-type virus, for replication [45,46], and have in other studies been associated with low viremia levels. This might, however, be dependent on genotype, since the strains in the former studies were of genotype C, while the strain with deletion in BCP in this study belonged to subgenotype A1 from an HBeAg positive individual, with a high viral load of HBV DNA, and without a known liver disease. No wild-type sequence was observed, but deep sequencing of the strains might be needed to identify low levels of wild-type virus. The strain with an 11bp insertion in the BCP region was also from an HBeAg positive individual, without a known liver disease. This insertion had a binding site for HNF1, and has been described previously in transplanted patients with fulminant hepatitis [23]. These results indicated that this and other BCP mutations that have been associated with disease aggravations, might be more pathogenic, only in certain viral strains or host settings.

## 5. Conclusions

This study showed that previously rarely described HBV variants expressing little or no HBeAg were selected among subgenotype Al strains, from carriers in Rwanda, after they had seroconverted to anti-HBe, and that these mutations might be clade specific. The most common mutations concerned the precore ORF, with either an altered start codon or a Kozak sequence. The study also confirmed the uniqueness of HBV in Rwanda, where almost all strains from every region of the country belonged to the same clade, within the subgenotype A1 [7]. The strains originated from all regions of Rwanda, and the low divergence between them indicates, either a recent introduction of HBV into Rwanda, with a rather rapid spread, or that this strain is more viremic than other introduced strains and, thereby, has a faster spread. No complete HBV genome was available from neighboring Burundi, but two strains from Tanzania and the Democratic Republic of the Congo were found in the Rwandan clade, indicating that the Rwandan strain might either have been imported from or exported to one of these countries.

## Figures and Tables

**Table 1:** HBeAg and anti-HBe in healthy persons and liver disease patients infected with sequenced subgenotype A1 strains. The number of complete genomes is given in parenthesis.

	*N*	HBeAg	Anti-HBe	Both HBeAg and Anti-HBe	No HBe Marker
No known liver disease	25 (14)	9 (7)	14 (5)	2 (2)	0
Liver disease patients	16 (9)	6 (5)	8 (3)	1 (1)	1 (0)
TOTAL	41 (23)	15 (12)	22 (8)	3 (3)	1 (0)

**Table 2:** Number of strains with different mutations in the BC, in relation to viral load and HBeAg/anti-HBe reactivity of the patients.

	All		A1762T/G1764A		Changed Kozak Sequence ^a^		Precore Start Codon Mutation	
	*N*	Viral Load^b^	*N*	Viral Load ^b^	*N*	Viral Load ^b^	*N*	Viral Load ^b^
*No known liver disease*								
HBeAg	9	9.27						
Anti-HBe	14	4.55	2	3.91	6	4.53	5	4.68
Both HBe markers	2	6.80	1	7.10				
*Subtotal*	*25*		*3*		*6*		*5*	
*Liver disease patients*								
HBeAg	6	7.43						
Anti-HBe	8	6.61	2	5.79	2	7.08	4^c^	5.50
Both HBe markers	1	5.43						
No HBe marker	1	3.11	1	3.11				
*Subtotal*	*16*		*3*		*2*		*4*	
*All samples*								
HBeAg	15	9.07						
Anti-HBe	22	6.20	4	5.49	8	6.48	9	5.22
Both HBe markers	3	6.64	1	7.10				
No HBe marker	1	3.11	1	3.11				
**TOTAL**	**41**		**6**	**6.10**	**8**	**6.48**	**9**	**5.22**

^a^ nt 1809–1813; ^b^ log HBV DNA copies/mL serum; ^c^ one with also the A1762T/G1764A double mutation.

**Table 3:** Number of strains sequenced in this study and identified from GenBank with different nucleotides at the start codon for pre-C, between positions 1814–1816.

	N Strains with Respective Precore Start Codons					
	AUG	CUG	ACG	UUG	AAG	AUA
*Without known liver disease*						
HBeAg reactive	9	0	0	0	0	0
Anti-HBe reactive	9	2	0	2	1	0
Both markers	2	0	0	0	0	0
*Subtotal*	***20***	***2***	***0***	***2***	***1***	***0***
*With known liver disease*						
HBeAg reactive	6	0	0	0	0	0
Anti-HBe reactive	4	1	1	1	1	0
Both markers	1	0	0	0	0	0
No HBe marker	1	0	0	0	0	0
*Subtotal*	***12***	***1***	***1***	***1***	***1***	***0***
*All patients*						
HBeAg reactive	15	0	0	0	0	0
Anti-HBe reactive	13	3	1	3	2	0
Both markers	3	0	0	0	0	0
No HBe marker	1	0	0	0	0	0
TOTAL	**32**	**3**	**1**	**3**	**2**	**0**
A1 sequences in GenBank	237	21	2	6	0	0
A2 sequences in GenBank	218	3	2	1	0	1
A3 sequences in GenBank	37	8	1	3	0	0

**Table 4:** Different changes in the Kozak sequence preceding the precore start codon in strains from patients infected with the A1 strains common in Rwanda and in sequences retrieved from the GenBank.

	N Strains with Respective Kozak Sequence At positions 1809–1813							Other Kozak Sequences
	A1 wild Type TCATC/ GCACC	TGGTC	TTCTC	TACTC	TCTTC	TCCTC	TCTGC	
*Without known liver disease*								
HBeAg reactive	9	0	0	0	0	0	0	
Anti-HBe reactive	9	0	2	1	1	1	0	
Both markers	1	1	0	0	0	0	0	
*Subtotal*	*19*	*1*	*2*	*1*	*1*	*1*	*0*	
*With known liver disease*								
HBeAg reactive	6	0	0	0	0	0	0	
Anti-HBe reactive	6	0	0	0	0	1	1	
Both markers	1	0	0	0	0	0	0	
No HBe marker	1	0	0	0	0	0	0	
*Subtotal*	*14*	*0*	*0*	*0*	*0*	*1*	*1*	
*All patients*								
HBeAg reactive	15	0	0	0	0	0	0	
Anti-HBe reactive	15	0	2	1	1	2	1	
Both markers	2	1	0	0	0	0	0	
No HBe marker	1	0	0	0	0	0	0	
***TOTAL***	***33***	***1***	***2***	***1***	***1***	***2***	***1***	
A1 sequences in GenBank	225	0	3	0	4	21	0	13
A2 sequences in GenBank	220	0	0	0	0	0	0	5
A3 sequences in GenBank	35	0	1	0	3	9	0	1

## Supplementary Materials

The following are available online at https://www.mdpi.com/2073-4425/10/3/182/s1, Figure S1: Branch from a UPGMA tree, based on 526 complete HBV genomes. The clades formed by each subgenotype A are shown in the small tree to the left. The branch formed by subgenotype A3 complete HBV genomes is enlarged, Table S1: Primers used for amplification and sequencing complete HBV genomes. Table S2: Designation, genome length, and position and nucleotide length of the 5 ORFs, in the sequenced complete HBV genomes.

## References

[1] Norder H., Courouce A.M., Coursaget P., Echevarria J.M., Lee S.D., Mushahwar I.K., Robertson B.H., Locarnini S., Magnius L.O. (2004). Genetic diversity of hepatitis B virus strains derived worldwide: Genotypes, subgenotypes, and HBsAg subtypes. Intervirology.

[2] Hannoun C., Norder H., Lindh M. (2000). An aberrant genotype revealed in recombinant hepatitis B virus strains from Vietnam. J. Gen. Virol..

[3] Olinger C.M., Jutavijittum P., Hubschen J.M., Yousukh A., Samountry B., Thammavong T., Toriyama K., Muller C.P. (2008). Possible new hepatitis B virus genotype, southeast Asia. Emerg. Infect. Dis..

[4] Tran T.T., Trinh T.N., Abe K. (2008). New complex recombinant genotype of hepatitis B virus identified in Vietnam. J. Virol..

[5] Kramvis A., Kew M.C. (2007). Epidemiology of hepatitis B virus in Africa, its genotypes and clinical associations of genotypes. Hepatol. Res. Off. J. Jpn. Soc. Hepatol..

[6] Kramvis A. (2014). Genotypes and genetic variability of hepatitis B virus. Intervirology.

[7] Twagirumugabe T., Swaibu G., Walker T.D., Lindh M., Gahutu J.B., Bergstrom T., Norder H. (2017). Hepatitis B virus strains from Rwandan blood donors are genetically similar and form one clade within subgenotype A1. BMC Infect. Dis.

[8] Tong S., Revill P. (2016). Overview of hepatitis B viral replication and genetic variability. J. Hepatol..

[9] Miller R.H., Kaneko S., Chung C.T., Girones R., Purcell R.H. (1989). Compact organization of the hepatitis B virus genome. Hepatology.

[10] Nassal M., Schaller H. (1993). Hepatitis B virus replication. Trends Microbiol..

[11] Magnius L.O., Espmark J.A. (1972). New specificities in Australia antigen positive sera distinct from the Le Bouvier determinants. J. Immunol..

[12] Milich D.R., Jones J.E., Hughes J.L., Price J., Raney A.K., McLachlan A. (1990). Is a function of the secreted hepatitis B e antigen to induce immunologic tolerance in utero?. Proc. Natl. Acad. Sci. USA.

[13] Chen M.T., Billaud J.N., Sallberg M., Guidotti L.G., Chisari F.V., Jones J., Hughes J., Milich D.R. (2004). A function of the hepatitis B virus precore protein is to regulate the immune response to the core antigen. Proc. Natl. Acad. Sci. USA.

[14] Okamoto H., Tsuda F., Akahane Y., Sugai Y., Yoshiba M., Moriyama K., Tanaka T., Miyakawa Y., Mayumi M. (1994). Hepatitis B virus with mutations in the core promoter for an e-antigen-negative phenotype in carriers with antibody to e antigen. J. Virol..

[15] Buckwold V.E., Xu Z., Chen M., Yen T.S., Ou J.H. (1996). Effects of a naturally occurring mutation in the hepatitis B virus basal core promoter on precore gene expression and viral replication. J. Virol..

[16] Chan H.L., Hussain M., Lok A.S. (1999). Different hepatitis B virus genotypes are associated with different mutations in the core promoter and precore regions during hepatitis B e antigen seroconversion. Hepatology.

[17] Chan H.L., Leung N.W., Hussain M., Wong M.L., Lok A.S. (2000). Hepatitis B e antigen-negative chronic hepatitis B in Hong Kong. Hepatology.

[18] Moriyama K., Okamoto H., Tsuda F., Mayumi M. (1996). Reduced precore transcription and enhanced core-pregenome transcription of hepatitis B virus DNA after replacement of the precore-core promoter with sequences associated with e antigen-seronegative persistent infections. Virology.

[19] Fiordalisi G., Cariani E., Mantero G., Zanetti A., Tanzi E., Chiaramonte M., Primi D. (1990). High genomic variability in the pre-C region of hepatitis B virus in anti-HBe, HBV DNA-positive chronic hepatitis. J. Med. Virol..

[20] Parekh S., Zoulim F., Ahn S.H., Tsai A., Li J., Kawai S., Khan N., Trepo C., Wands J., Tong S. (2003). Genome replication, virion secretion, and e antigen expression of naturally occurring hepatitis B virus core promoter mutants. J. Virol..

[21] Nishizawa T., Hoshino T., Naganuma A., Kobayashi T., Nagashima S., Takahashi M., Takagi H., Okamoto H. (2016). Enhanced pregenomic RNA levels and lowered precore mRNA transcription efficiency in a genotype A hepatitis B virus genome with C1766T and T1768A mutations obtained from a fulminant hepatitis patient. J. Gen. Virol..

[22] Laskus T., Rakela J., Tong M.J., Nowicki M.J., Mosley J.W., Persing D.H. (1994). Naturally occurring hepatitis B virus mutants with deletions in the core promoter region. J. Hepatol..

[23] Pult I., Chouard T., Wieland S., Klemenz R., Yaniv M., Blum H.E. (1997). A hepatitis B virus mutant with a new hepatocyte nuclear factor 1 binding site emerging in transplant-transmitted fulminant hepatitis B. Hepatology.

[24] Tong S.P., Li J.S., Vitvitski L., Trepo C. (1990). Active hepatitis B virus replication in the presence of anti-HBe is associated with viral variants containing an inactive pre-C region. Virology.

[25] Carman W.F., Jacyna M.R., Hadziyannis S., Karayiannis P., McGarvey M.J., Makris A., Thomas H.C. (1989). Mutation preventing formation of hepatitis B e antigen in patients with chronic hepatitis B infection. Lancet.

[26] Okamoto H., Yotsumoto S., Akahane Y., Yamanaka T., Miyazaki Y., Sugai Y., Tsuda F., Tanaka T., Miyakawa Y., Mayumi M. (1990). Hepatitis B viruses with precore region defects prevail in persistently infected hosts along with seroconversion to the antibody against e antigen. J. Virol..

[27] Santantonio T., Jung M.C., Miska S., Pastore G., Pape G.R., Will H. (1991). Prevalence and type of pre-C HBV mutants in anti-HBe positive carriers with chronic liver disease in a highly endemic area. Virology.

[28] Raimondo G., Schneider R., Stemler M., Smedile V., Rodino G., Will H. (1990). A new hepatitis B virus variant in a chronic carrier with multiple episodes of viral reactivation and acute hepatitis. Virology.

[29] Twagirumugabe T.M.C., Habarurema S., Seruyange E., Bergström T., Gahutu J.B., Walker T.D., Norder H. (2018). Different Epidemiology of Two Blood Borne Infections, Hepatitis B and C, in Rwanda.

[30] Radziwill G., Tucker W., Schaller H. (1990). Mutational analysis of the hepatitis B virus P gene product: Domain structure and RNase H activity. J. Virol..

[31] Kimbi G.C., Kramvis A., Kew M.C. (2004). Distinctive sequence characteristics of subgenotype A1 isolates of hepatitis B virus from South Africa. J. Gen. Virol..

[32] Kozak M. (1997). Recognition of AUG and alternative initiator codons is augmented by G in position +4 but is not generally affected by the nucleotides in positions +5 and +6. EMBO J..

[33] Ahn S.H., Kramvis A., Kawai S., Spangenberg H.C., Li J., Kimbi G., Kew M., Wands J., Tong S. (2003). Sequence variation upstream of precore translation initiation codon reduces hepatitis B virus e antigen production. Gastroenterology.

[34] Chen I.H., Huang C.J., Ting L.P. (1995). Overlapping initiator and TATA box functions in the basal core promoter of hepatitis B virus. J. Virol..

[35] Kimbi G.C., Kew M.C., Kramvis A. (2012). The effect of the G1888A mutation of subgenotype A1 of hepatitis B virus on the translation of the core protein. Virus Res..

[36] Ochwoto M., Chauhan R., Gopalakrishnan D., Chen C.Y., Ng’ang’a Z., Okoth F., Kioko H., Kimotho J., Kaiguri P., Kramvis A. (2013). Genotyping and molecular characterization of hepatitis B virus in liver disease patients in Kenya. Infect. Genet. Evol..

[37] Andernach I.E., Nolte C., Pape J.W., Muller C.P. (2009). Slave trade and hepatitis B virus genotypes and subgenotypes in Haiti and Africa. Emerg. Infect. Dis..

[38] Hubschen J.M., Mbah P.O., Forbi J.C., Otegbayo J.A., Olinger C.M., Charpentier E., Muller C.P. (2011). Detection of a new subgenotype of hepatitis B virus genotype A in Cameroon but not in neighbouring Nigeria. Clin. Microbiol. Infect..

[39] Touriol C., Bornes S., Bonnal S., Audigier S., Prats H., Prats A.C., Vagner S. (2003). Generation of protein isoform diversity by alternative initiation of translation at non-AUG codons. Biol. Cell.

[40] Dever T.E. (2012). Molecular biology. A new start for protein synthesis. Science.

[41] Norder H., Galli C., Magnil E., Sikora P., Ekvarn E., Nystrom K., Magnius L.O. (2018). Hepatitis E virus genotype 3 genomes from RNA-positive but serologically negative plasma donors have CUG as the start codon for ORF3. Intervirology.

[42] Firth A.E., Brierley I. (2012). Non-canonical translation in RNA viruses. J. Gen. Virol..

[43] Li J.S., Tong S.P., Wen Y.M., Vitvitski L., Zhang Q., Trepo C. (1993). Hepatitis B virus genotype A rarely circulates as an HBe-minus mutant: Possible contribution of a single nucleotide in the precore region. J. Virol..

[44] Wei F., Zheng Q., Li M., Wu M. (2017). The association between hepatitis B mutants and hepatocellular carcinoma: A meta-analysis. Medicine.

[45] Li K.S., Yamashiro T., Sumie A., Terao H., Mifune K., Nishizono A. (2001). Hepatitis B virus harboring nucleotide deletions in the core promoter region and genotype B correlate with low viral replication activity in anti-HBe positive carriers. J. Clin. Virol. Off. Publ. Pan Am. Soc. Clin. Virol..

[46] Kohno K., Nishizono A., Terao H., Hiraga M., Mifune K. (2000). Reduced transcription and progeny virus production of hepatitis B virus containing an 8-bp deletion in basic core promoter. J. Med. Virol..

