# Supplementary material for: High Frequency of Either Altered Pre-Core Start Codon or Weakened Kozak Sequence in the Core Promoter Region in Hepatitis B Virus A1 Strains from Rwanda
Source: Genes (Basel). 2019 Feb 26;10(3):182. doi: 10.3390/genes10030182 (PMC6471190; doi:10.3390/genes10030182)

Table S1. Primers used for amplification and sequencing of complete HBV genomes.

Primer designation	Primer sequence
**B-464S1**	TTGCCCGTTTGTCCTCTAATTCCAGGATCC
**B-464S2**	TTGCCCGTTTGTCCTCTSATTCCAGGATCC
**P2**	AAAAAGTTGCATGRTGMTGG
**P2L2**	CCGGAAAGCTTGAGCTCTTCAAAAAGTTGCATGGTGCTGG
**595F**	CAC HTG TAT TCC CAT CCC A
**179R**	CCA ATT TMT GCY TAC AGC CTC
**1584F**	ACT TCG MBT CAC CTC TGC ACG T
**2396R**	GTC KGC GAG GYG AGG GAG TT
**2331R**	GGA AGY GTK GAY ARG ATA GGG GCA TT
**1859F**	ACT NTT CAA GCC TCC RAG CTG
**2835R**	GTT CCC AVG WAT AWG GTG AYC C
**1877F**	CTG TGC CTT GGR TGG CTT
**gtA1-2792S1**	GGAAACTACACGTAGCGCCTCATTTTG
**gtA1-2792S2**	GGAAACTACACGTAGCGCTTCATTTTG
**B-874R1**	ACCCCAACTTCCAATTACATAKCCCATGAA
**B-874R2**	RCCCCAACTTCCAATTATRTATCCCATGAA
**B-874R4**	GCCCCAACTTCCAATYACATATCCcATGAA
**B-548R1**	TTTTGTACAGCAACATGRGGGAAACATAGA
**B-548R2**	TTTTGTACAGCAACATGAGGGAATCATAGA
**B-98R1**	TTGACGAGATGTGAGAGGCARTATT
**B-98R2**	TTGACGATATGTGAGAGACAATATT
**B-98R3**	TTGACGATATGTGAGAGGCAATATT
**B-98R4**	TGGAGGAGATGGGAGAGGCAATATT

Table S2. Designation, genome length and position, and nucleotide length of the 5 ORFs in the sequenced complete HBV genomes.

Strains designation	Complete genomes N nucleotides	P gene (2307-1623) N nucleotides Deletions positions	S gene (2854-835)	Pre-C gene (1814-1900) N nucleotides (altered start codon)	C gene (1901-2458) N nucleo-tides	X gene (1374-1838)
**Rw14-03**	3167	2484 del 1-54	1149	87	552	465
**Rw14-14**	3167	2484 del 1-54	1149	87	558	465
**Rw14-25**	3221	2538	1203	87	558	465
**Rw14-37**	3221	2538	1170	87	558	465
**Rw14-48**	3221	2538	1203	87	558	465
**Rw14-65**	3221	2538	1203	87	558	465
**Rw14-120**	3221	2538	1203	87 (TTG)	558	465
**Rw14-126**	3221	2538	1203	87	558	465
**Rw14-136**	3167	2484 del 1-54	1149	87	558	465
**Rw14-203**	3221	2538	1203	87	558	465
**Rw14-220**	3233	2538	1203	87	558	408 12 nt insert between 1766 and 1777
**Rw14-226**	3158	2475 del 3051-3113	1140	87	558	465
**Rw2003**	3221	2538	1203	87	558	465
**Rw2016**	3221	2538	1203	87	558	465
**Rw2079**	3200	2517 del 1573-1590 del 3160-3163	1200	87	558	447 18 nt deletion between 1573 and 1590
**Rw2086**	3188	2505 del 22-54	1170	87	558	465
**Rw2113**	3221	2538	1203	87 (AAG)	558	465
**Rw2157**	3170	2487 del 1-51	1152	87 (TTG)	558	465
**Rw2199**	3182	2538	1203	87	558	426 39 nt deletion between 1736 and 1775
**Rw2226**	3167	2484 del 1-54	1149	87	558	465
**Rw2244**	3167	2484 del 1-54	1149	87	558	465
**Rw3173**	3221	2538	1203	87	558	465
**Rw3531**	3221	2538	1203	87	558	465

Figure S1. Branch from a UPGMA tree, based on 526 complete HBV genomes. The clades formed by each subgenotype of A are shown in the small tree to the left. The branch formed by subgenotype A3 complete HBV genomes is enlarged. The origin of strains from the same country is marked by brackets at the nodes, the origin of the other stains are given at the nodes. Strains with wild-type precore start codon and Kozak sequence preceding the precore start from patients with unknown HBeAg/anti-HBe status are marked by black squares at the nodes. Strains from HBeAg patients are marked by green squares. The strains marked with red or orange squares have either a changed precore start codon (red) or a changed Kozak sequence (orange). The figures below the branches refer to the bootstrap values of 1000 replicas.

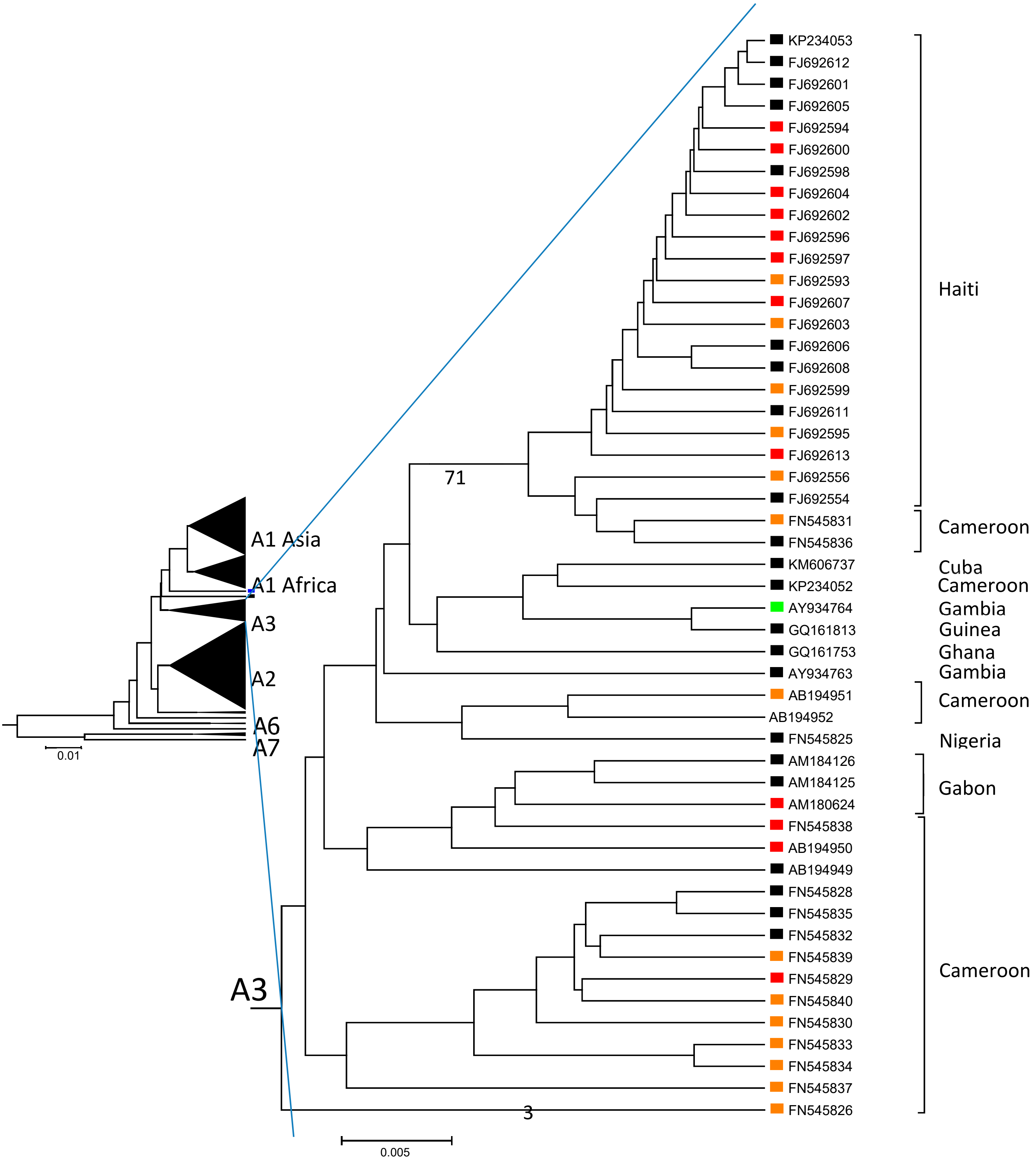

Supplement: Supplementary file 1 [file genes-10-00182-s001.pdf]
